# CEP55: an immune-related predictive and prognostic molecular biomarker for multiple cancers

**DOI:** 10.1186/s12890-023-02452-1

**Published:** 2023-05-12

**Authors:** Guo-Sheng Li, Wei Zhang, Wan-Ying Huang, Rong-Quan He, Zhi-Guang Huang, Xiang-Yu Gan, Zhen Yang, Yi-Wu Dang, Jin-Liang Kong, Hua-Fu Zhou, Gang Chen

**Affiliations:** 1 Department of Cardiothoracic Surgery, The First Affiliated Hospital of Guangxi Medical University, Guangxi Zhuang Autonomous Region, No. 6, Shuangyong Road, 530021 Nanning, P. R. China; 2 Department of Pathology, The First Affiliated Hospital of Guangxi Medical University, Guangxi Zhuang Autonomous Region, No. 6, Shuangyong Road, 530021 Nanning, P. R. China; 3 Department of Oncology, The First Affiliated Hospital of Guangxi Medical University, Guangxi Zhuang Autonomous Region, No. 6, Shuangyong Road, 530021 Nanning, P. R. China; 4 Division of Pulmonary and Critical Care Medicine, The First Affiliated Hospital of Guangxi Medical University, No. 6, Shuangyong Road, 530021 Nanning, Guangxi P. R. China

**Keywords:** Centrosomal protein 55, Cancer biology, Cancer genetics, Prognosis, Biomarker

## Abstract

**Background:**

Centrosomal protein 55 (*CEP55*) plays a significant role in specific cancers. However, comprehensive research on *CEP55* is lacking in pan-cancer.

**Methods:**

In-house and multi-center samples (*n* = 15,823) were used to analyze *CEP55* in 33 cancers. The variance of *CEP55* expression levels among tumor and control groups was evaluated by the Wilcoxon rank-sum test and standardized mean difference (SMD). The clinical value of *CEP55* in cancers was assessed using receiver operating characteristic (ROC) curves, Cox regression analysis, and Kaplan-Meier curves. The correlations between *CEP55* expression and the immune microenvironment were explored using Spearman’s correlation coefficient.

**Results:**

The data of clustered regularly interspaced short palindromic repeats confirmed that *CEP55* was essential for the survival of cancer cells in multiple cancer types. Elevated *CEP55* mRNA expression was observed in 20 cancers, including glioblastoma multiforme (*p* < 0.05). *CEP55* mRNA expression made it feasible to distinguish 21 cancer types between cancer specimens and their control samples (AUC = 0.97), indicating the potential of *CEP55* for predicting cancer status. Overexpression of *CEP55* was correlated with the prognosis of cancer individuals for 18 cancer types, exhibiting its prognostic value. *CEP55* expression was relevant to tumor mutation burden, microsatellite instability, neoantigen counts, and the immune microenvironment in various cancers (*p* < 0.05). The expression level and clinical relevance of *CEP55* in cancers were verified in lung squamous cell carcinoma using in-house and multi-center samples (SMD = 4.07; AUC > 0.95; *p* < 0.05).

**Conclusion:**

*CEP55* may be an immune-related predictive and prognostic marker for multiple cancers, including lung squamous cell carcinoma.

**Supplementary Information:**

The online version contains supplementary material available at 10.1186/s12890-023-02452-1.

## Background

Cancer is a common cause of human mortality and a severe obstacle to the goal of improving life expectancy worldwide. Cancer morbidity and mortality are growing globally, with over 19 million newly diagnosed individuals and more than 10 million deaths occurring in 2020 [1]. Traditional cancer treatments, such as radiation, chemotherapy, and targeted therapies, benefit cancer patients; however, some limitations of these treatments have been identified in recent years. For example, they may cause irreparable DNA damage, produce more off-target effects, and disrupt the tumor microenvironment [2–4]. For this reason, combining immunotherapy and conventional therapies has emerged as a powerful clinical strategy for cancer treatment. Thus, performing multi-center studies on various cancers is important for exploring potential biomarkers suitable for multiple cancers, as this may provide a basis for investigating cancer immunotherapy targets [5].

One promising target is the protein encoded by centrosomal protein 55 (*CEP55*) (also known as *CT111* and *C10orf3*). CEP55 is an essential component of the CEP family and an important factor regulating mitotic termination and cytoplasmic division, with localization at different locations important in the cell cycle, such as the centrosome or spindle [6, 7]. Previous reports have demonstrated elevated *CEP55* expression in breast cancer [8], liver cancer [9], colon cancer [10], and lung adenocarcinoma (LUAD) [11]. This upregulated expression has also shown significant correlations with stage, metastasis, and unfavorable clinical outcome in various tumors [7]. *CEP55* can affect the PI3K/AKT pathway and cell cycle, and the knockdown of *CEP55* can inhibit tumor cell viability and proliferation and can even lead to tumor cell death [12, 13]. Some studies have also reported that a high expression of *CEP55* promotes tumor development, metastasis, and aggression by activating the PI3K/AKT signal pathway [14]. Increasing evidence now suggests a clear association between *CEP55* upregulation and the development and progression of multiple malignancies. However, research on the involvement of *CEP55* in pan-cancer is lacking, and the clinical significance and potential mechanisms of this gene in multiple cancers require further elucidation.

This study is the first to focus on *CEP55* in pan-cancer. Public and in-house data were used to investigate the expression of CEP55 at the messenger RNA (mRNA) and protein levels, with the aim of revealing the conspicuous clinical relevance of *CEP55* in multiple cancers. An extensive investigation of lung squamous cell carcinoma (LUSC), based on internal tissue microarrays and multi-center samples, was also performed to validate the clinical significance of *CEP55* in cancers. In conclusion, the findings of this study, which focused on the importance of *CEP55* expression in various tumors, identified *CEP55* as a potential novel immune-related predictive and prognostic marker for multiple cancers.

## Materials and methods

### Collection of pan-cancer expression data

The Depmap Portal is an extensive database of missing cell function screens that can be used to study the underlying dependence of multiple genes in human cancer cell lines, thereby allowing assessment of the essential roles of those genes [15, 16]. For this study, a dataset (*n* of samples = 1,068) including CRISPR (clustered regularly interspaced short palindromic repeats) Chronos scores of CEP55 were downloaded from the Depmap Portal.

The Cancer Genome Atlas (TCGA) sample data, including 33 cancer types and their 21 control tissue types, were downloaded from the Xena database. Three sample categories – primary tumors, normal solid tissue, and primary hematogenous cancer peripheral blood – from the TCGA cohort were also included in this study, including 9,358 cancer samples and 722 control samples. Details of the 33 cancers in the TCGA cohort are listed in Table S1, which also provides the abbreviations for these cancers.

The pan-cancer protein level data (*n* of samples = 1,719) [17–23] used in this study originated from the Clinical Proteomic Tumor Analysis Consortium, available from Proteomic Data Commons (https://pdc.cancer.gov).

### Clinical relevance of ***CEP55*** expression in pan-cancer

CRISPR screens allow phenotypic screening and high-throughput sequencing analysis of target cells through the construction of small guide RNA libraries, leading to the exploitation of critical genes [24]. Chronos is a model of cell group dynamics in CRISPR knockout screens [25], and this study identified the critical role of *CEP55* in multiple cancers through Chronos. Data from the cell depletion assay can be indicated by the Chronos score, and a Chronos score of < 0 indicates that the gene is necessary for selected cell lines.

The capacity of *CEP55* expression to distinguish between tumor and control tissues can be ascertained using receiver operating characteristic (ROC) curves and summary ROC (sROC) curves for sensitivity, specificity, and area under the curve (AUC) values. Larger sensitivity, specificity, and AUC values indicate a more conspicuous ability of *CEP55* to identify cancers and their controls.

Prognosis is an essential clinical parameter for cancer patients. This study considered four clinical outcomes reflecting patient prognosis: overall survival (OS), disease-specific survival (DSS), progression-free interval (PFI), and disease-free interval (DFI). For single cancer, the correlation between *CEP55* expression and survival indicators for patients with that cancer was investigated using univariate Cox analysis and Kaplan-Meier curves. Cancer patients with various clinical features may have different prognoses; therefore, the relationship between *CEP55* expression and the clinical characteristics of the patients was also evaluated. Prognostic data and clinical characteristics (age, sex, and AJCC [American Joint Committee on Cancer] stage) for the above analysis were available from the Xena database.

### Data on tumor mutation burden, microsatellite instability, neoantigen number, and immune microenvironment

SangerBox (v3.0) [26] provides data related to tumor mutation burden (TMB), microsatellite instability (MSI) [27], and neoantigen counts [28]. The TIMER algorithm [29] can be applied to detect the level of infiltration by six immune cell types: B cells, CD4 + T cells, CD8 + T cells, neutrophils, macrophages, and dendritic cells. ESTIMATE [30, 31] reflects the immune environment of cancer patients by assigning three other scores: immune score, stromal score, and ESTIMATE score [32]. The ESTIMATE algorithm was used to calculate three scores to evaluate the correlation between *CEP55* and the immune microenvironment. The infiltration levels of the six immune cell types calculated by TIMER were available from the official TIMER website, while the three scores identified by ESTIMATE were downloaded from SangerBox (v3.0).

### Potential mechanisms of ***CEP55*** in multiple tumors

In this study, the “clusterProfiler” package [33] was applied to perform gene set enrichment analysis (GSEA) [34], with the aim of allowing further exploration of the potential involvement of CEP55 in signaling pathways associated with various cancers. The signaling pathways examined in this analysis were acquired from the Kyoto Encyclopedia of Genes and Genomes (KEGG) database [35–38].

### Research on ***CEP55*** in LUSC

The mRNA expression of *CEP55* in LUSC was explored using multi-center microarrays and RNA-Seq datasets [39, 40] from the ArrayExpress, Gene Expression Omnibus, TCGA, and GTEx [41] databases. Datasets were included using the following selection criteria: [1] microarrays or RNA-Seq; [2] tissue samples acquired from *Homo sapiens*; and [3] containing *CEP55* mRNA expression data. The standards for data exclusion were the following: [1] datasets with duplicate data and [2] fewer than three LUSC samples or control samples in merged datasets. This study covered 35 datasets containing 1,390 LUSC and 1,418 non-LUSC samples (Table S2). In addition, 288 LUSC cases from five datasets (GSE29013, GSE30219, GSE37745, GSE73403, and GSE81089) were obtained to analyze the possible association between *CEP55* expression with OS in LUSC patients.

The datasets available from public databases were standardized using log_2_ (*x* + 1) and the “limma” package [42]. Since each dataset was derived from a different batch of experiments, the original datasets were merged according to the same platform number using the “SVA” package [43] to eliminate batch effects [44]. For instance, the gene expression data from the GSE31552 and GSE44077 datasets were detected using the GPL6244 platform, leading to their consolidation into a merged cohort named GPL6244. In this study, the 35 datasets were divided into 11 new cohorts with the same platform numbers.

To clarify the differences in protein expression between LUSC and non-LUSC patients, immunohistochemistry (IHC) experiments were conducted on four in-house tissue microarrays (LUC481, LUC482, LUC483, and LUC1021). This study used a rabbit anti-human *CEP55* monoclonal antibody (EPR11944[B], ab170414, dilution ratio 1:100) and a second antibody-labeled horseradish peroxidase (D-3004-15, Changdao Biotechnology Co., Ltd., Shanghai, China). The steps of the IHC experiments and the protein score criteria were published previously [45].

### Statistical analysis

This study evaluated the variance of *CEP55* expression levels among tumor and control groups (e.g., LUSC vs. control) using the Wilcoxon rank-sum test and standardized mean difference (SMD). For the SMD, a random effects model was used when significant heterogeneity existed between the data sets, as determined by *I*^*2*^ values > 50% and a chi-square test *p*-value < 0.1. Otherwise, a fixed-effects model was applied. The outcomes of the SMD were statistically significant in specific cases (the corresponding 95% confidence interval [CI] excludes 0). *Begg*’s test was applied to evaluate the publication bias of the SMD, and a *p* value of less than 0.1 indicated a significant publication bias for SMD outcomes.

**Fig. 1:**
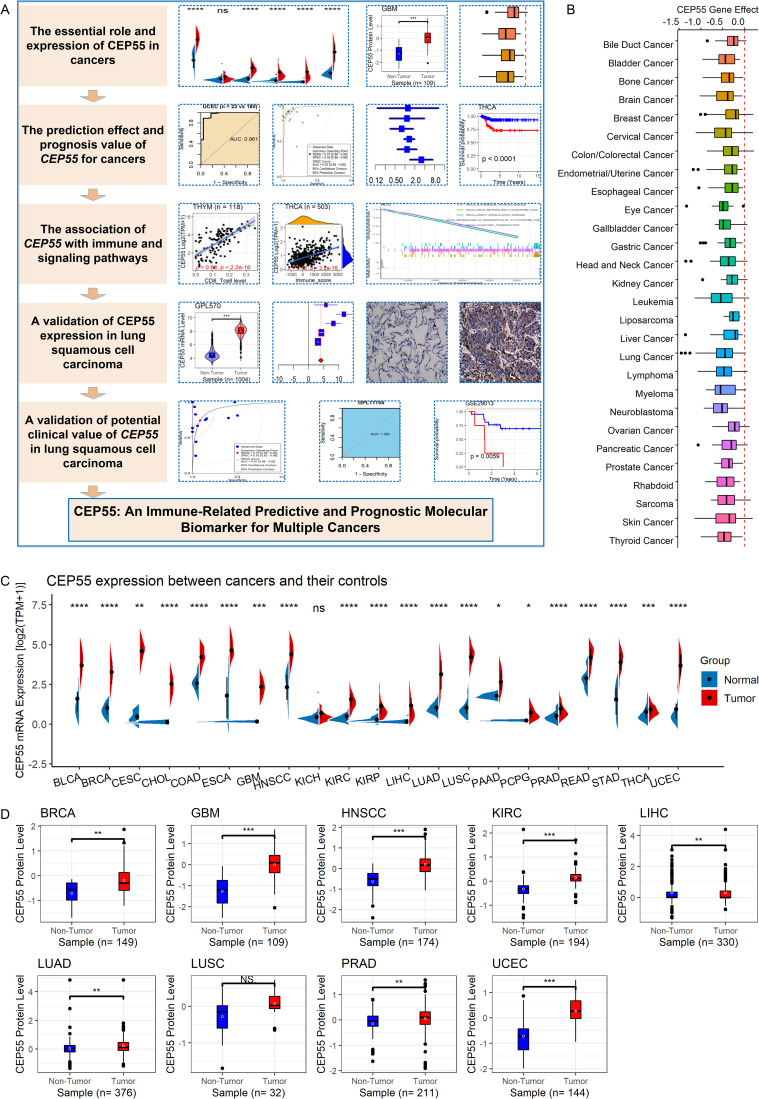
The research overflow, essential roles, and expression of CEP55 in cancers. Panel **A**: The research overflow of this study. Panel **B**: Identification of essential roles of *CEP55* for multiple cancers. Panel **C**: The differential expression of *CEP55* mRNA between cancers and controls; *p*-value was based on the Wilcoxon rank-sum test with a false discovery rate. Panel **D**: The differential levels of CEP55 protein between cancers and controls. ^ns/NS^*p* > 0.05; ^*^p < 0.05; ^**^p < 0.01; ^***^p < 0.001; ^****^p < 0.0001

The Wilcoxon rank-sum test was used to evaluate the correlation of *CEP55* expression with age, gender, and AJCC stage. Correlations among the *CEP55* expression and TMB, MSI, neoantigen count, TIMER score, and ESTIMATE score were analyzed using Spearman’s correlation coefficient, with *p* < 0.05 considered to indicate a significant statistical difference. For the hazard ratio (HR), 95% CI excluding 1 or *p* < 0.05 indicated statistical significance. Stata (v15.0) was used to plot the sROC, and R (v4.1.0) was used to complete all the remaining computation and visualization steps. The research overflow of this study is shown in Fig. 1A.

## Results

### Expression of CEP55 and its essential role in various cancers

The Chronos score reflected the importance of *CEP55* in various cancer cell lines, and a value of <0 indicated that decreased expression of *CEP55* increased cancer cell death. According to Fig. 1B, the Chronos scores of *CEP55* in the majority of 28 cancer cell lines were less than 0, indicating that *CEP55* was essential for tumor cells in some organs, such as bladder cancer, bone cancer, and gastric cancer.

Among the 21 cancers explored, *CEP55* mRNA was overexpressed in 20 cancer tissues (except kidney chromophobe [KICH]) when compared with the respective normal tissues (*p* < 0.05; Fig. 1C). CEP55 protein expression data for nine of the 20 cancers available from the Clinical Proteomic Tumor Analysis Consortium were collected for this research. The nine cancers were BRCA, GBM, HNSCC, KIRC, LIHC, LUAD, LUSC, PRAD, and UCEC. CEP55 protein levels were higher in cancer tissues than in normal tissues in eight of the nine cancers (except for LUSC) (Fig. 1D; *p* < 0.05), and the findings were consistent with the conclusions corroborating the results at the mRNA level.

### Clinical significance of ***CEP55*** expression in pan-cancer

**Fig. 2:**
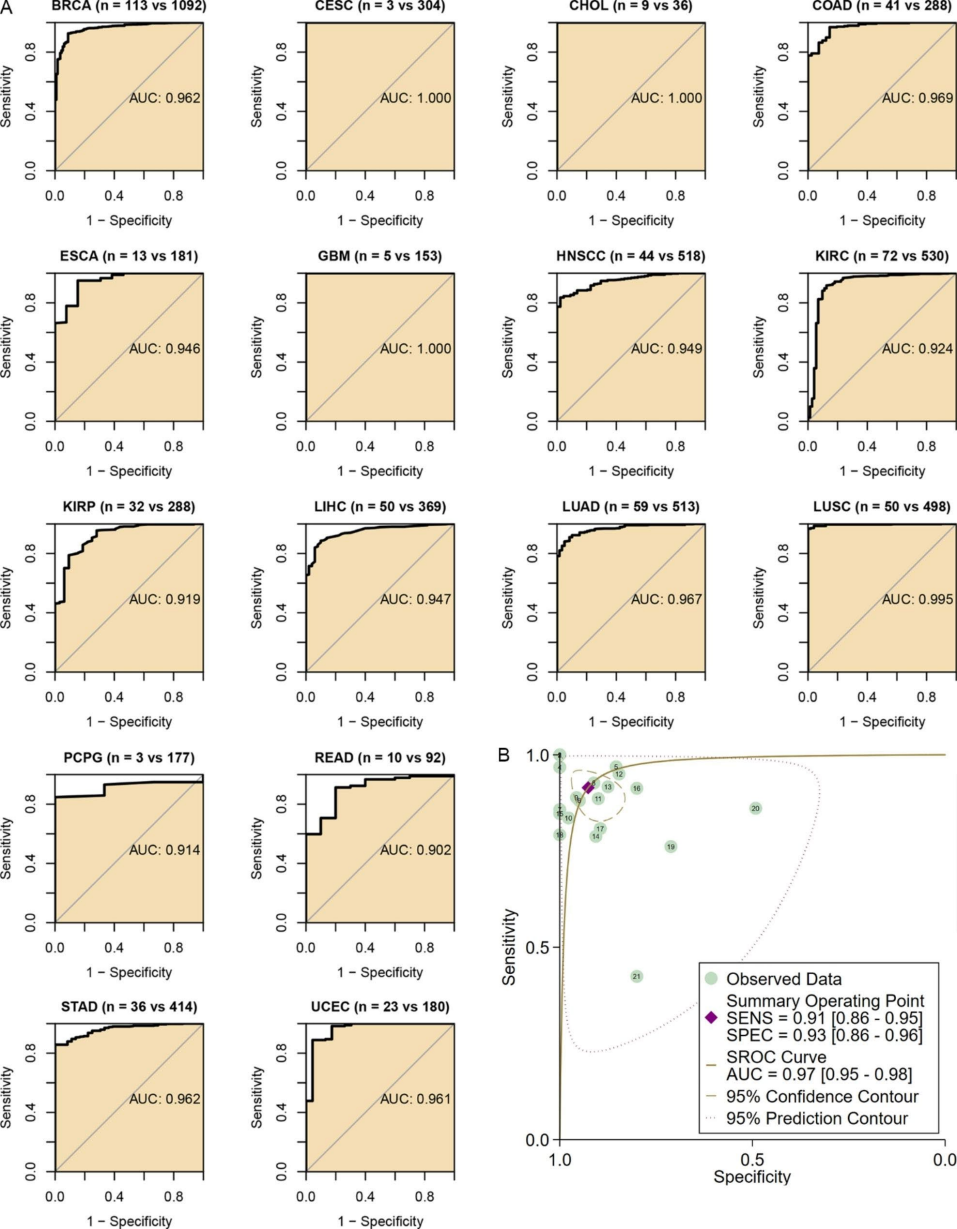
The ability of *CEP55* to differentiate the tumor tissue from control tissue. Panel **A**: *CEP55* can accurately distinguish cancer tissues from control tissues in some cancers. Panel **B**: *CEP55* distinguishes cancers well from control tissues in 21 cancer types

Given the essential roles and distinct expression levels of CEP55 in a wide range of cancers, the clinical relevance of *CEP55* in multiple cancers was further investigated. Sixteen of 21 cancer types had an AUC value of > 0.9 for *CEP55* expression, indicating a significant ability of *CEP55* expression to distinguish these 16 cancer tissues from their control tissues (Fig. 2A). For the 21 investigated cancers, the results of the sROC analysis showed that *CEP55* expression was highly accurate in identifying 21 types of cancers (sensitivity = 0.91, specificity = 0.93, AUC = 0.97; Fig. 2B).

**Fig. 3:**
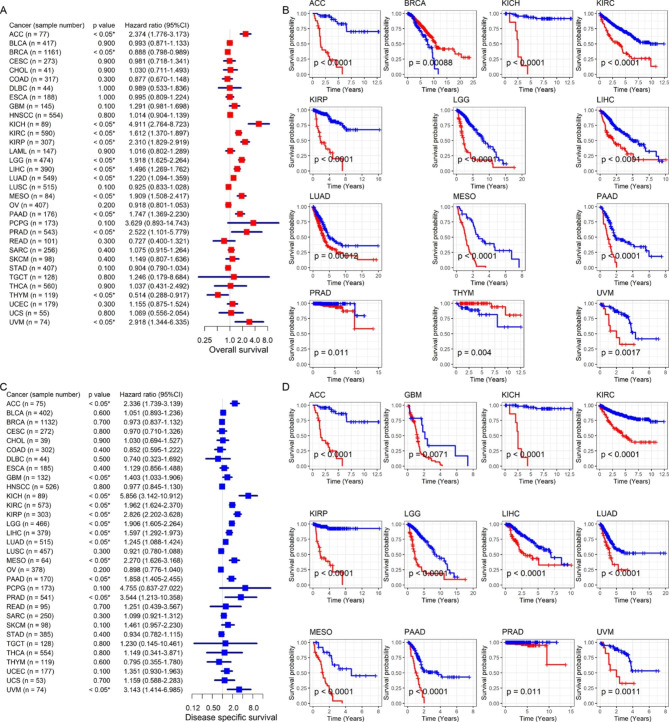
Relation of *CEP55* expression with overall survival and disease-specific survival of cancer patients. Panels **A** and **C**: *CEP55* expression was related to the prognosis of patients in most cancers. Panels **B** and **D**: *CEP55* expression was related to the prognosis of patients in some cancers; the red curve represents the high-*CEP55* expression group, while the blue curve represents the low-*CEP55* expression group. ^*^p < 0.05

**Fig. 4:**
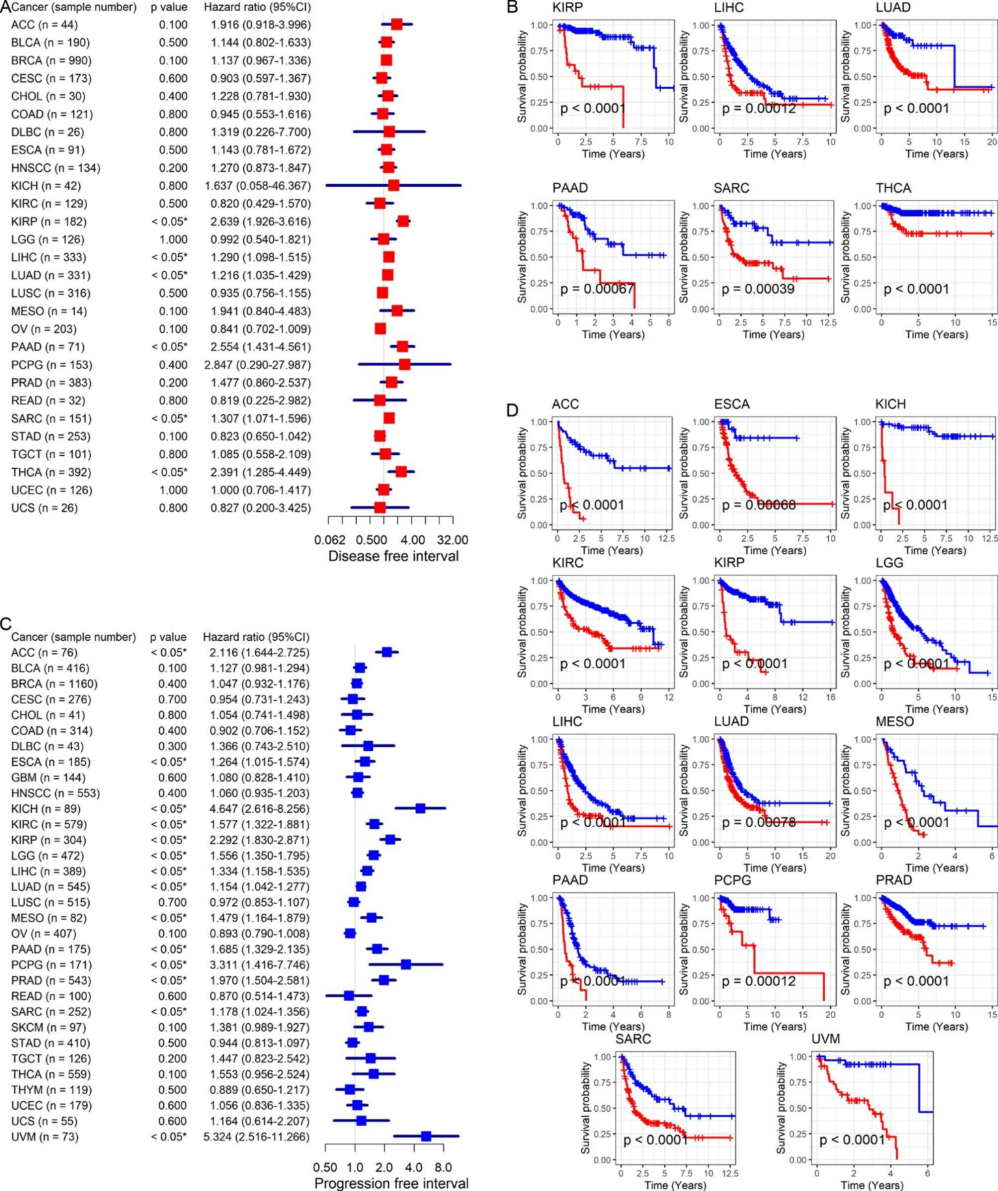
Relation of *CEP55* expression with disease-free interval and progression-free interval of cancer patients. Panels **A** and **B**: *CEP55* expression was related to the prognosis of patients in most cancers. Panels **B** and **D**: *CEP55* expression was related to the prognosis of patients in some cancers; the red curve represents the high-*CEP55* expression group, while the blue curve represents the low-*CEP55* expression group. ^*^p < 0.05

The study also explored the prognostic value of *CEP55* in distinct cancers. Upregulation of *CEP55* was associated with poor OS and DSS in patients with ACC, KICH, KIRC, KIRP, LGG, LIHC, LUAD, MESO, PAAD, PRAD, and UVM (HR > 1, *p* < 0.05; Fig. 3A–D). Elevated *CEP55* expression also indicated unfavorable DSS results in GBM patients (HR > 1, *p* < 0.05; Fig. 3 C–D). By contrast, in patients with BRCA or THYM, upregulated *CEP55* expression implied that they had longer OS (HR < 1, *p* < 0.05; Fig. 3A–B). Furthermore, high *CEP55* expression suggested shorter DFI for patients with certain cancers (KIRP, LIHC, LUAD, PAAD, SARC, and THCA) and shorter PFI for those suffering from ACC, ESCA, KICH, KIRC, KIRP, LGG, LIHC, LUAD, MESO, PAAD, PCPG, PRAD, SARC, and UVM (HR > 1, *p* < 0.05; Fig. 4A–D). Notably, upregulated *CEP55* expression represented poor prognostic indicators (i.e., OS, DSS, DFI, and PFI) for patients with one of four cancers: KIRP, LIHC, LUAD, and PAAD.

### Correlation of ***CEP55*** expression with clinical features

**Fig. 5:**
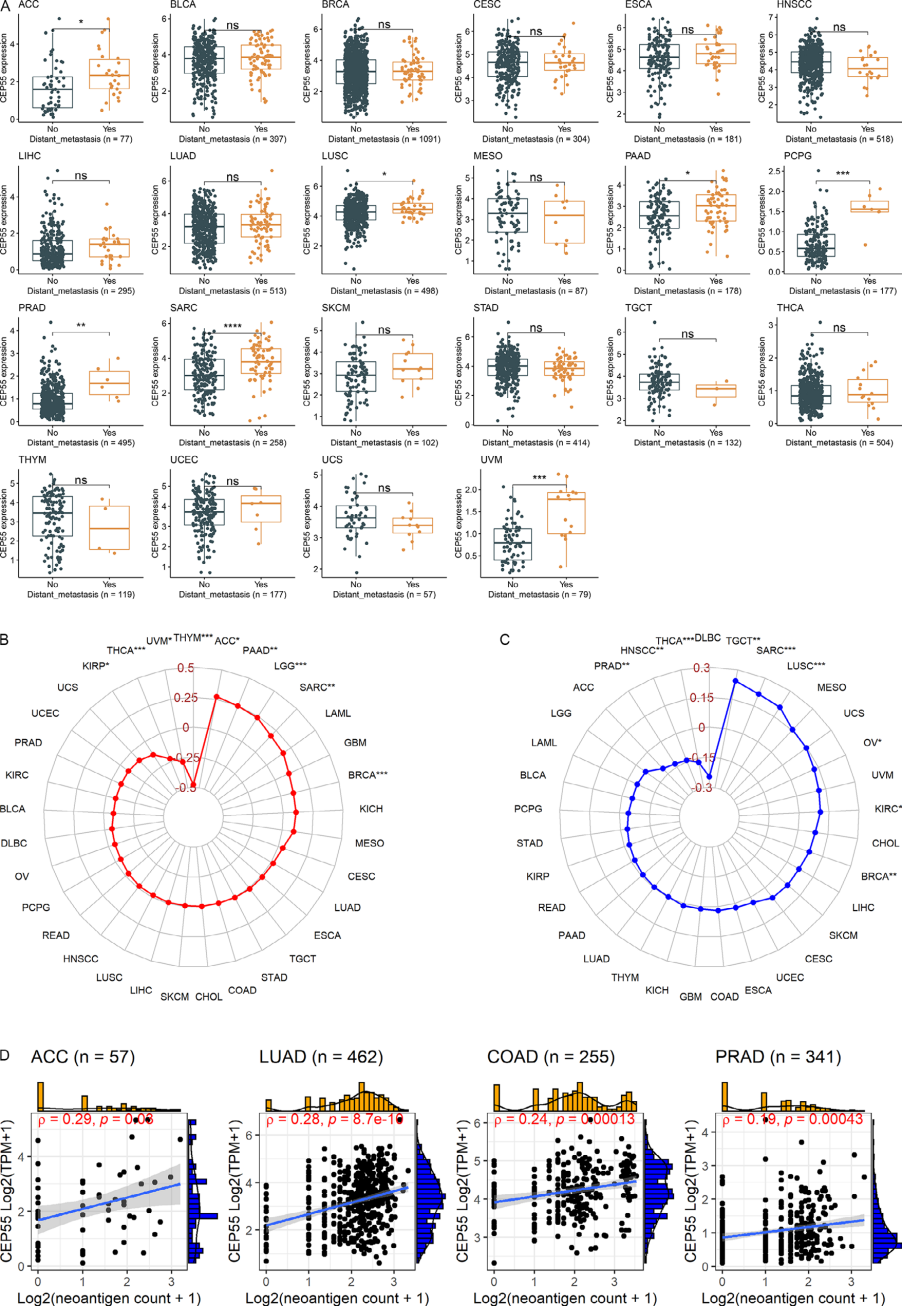
Relation of *CEP55* expression with tumor distant metastasis (panel **A**), tumor mutational burden (panel **B**), microsatellite instability (panel **C**), and neoantigen (panel **D**) of cancer patients. ^ns^*p* > 0.05; ^*^p < 0.05; ^**^p < 0.01; ^***^p < 0.001; ^****^p < 0.0001

As shown above, *CEP55* may be a prognostic marker for numerous cancers. Therefore, the association between *CEP55* expression levels and clinical characteristics (e.g., distant metastasis) of cancer patients was examined. Fig. 5 A reveals higher *CEP55* expression levels in patients with distant metastasis than in those without for seven cancers: ACC, LUSC, PAAD, PCPG, PRAD, SARC, and UVM (*p* < 0.05). Notably, *CEP55* emerged as a prognostic risk factor in almost all seven of these cancers, as depicted in Figs. 3 and 4. Thus, both results verified each other. In contrast to the aforementioned cancers, a statistical difference was not noted in *CEP55* expression levels for patients with distinct metastasis status in the remaining 15 cancers (Fig. 5A). Similar results were observed for different AJCC stages, ages, and genders (Fig. S1–S3). These findings suggest that, for most cancers, *CEP55* expression is not affected by the distant metastasis status, AJCC stages, ages, or genders of cancer patients and can be considered an independent marker.

### Relevance of ***CEP55*** expression to TMB, MSI, neoantigen count, and immune microenvironment

TMB is recognized as a potential prognostic biomarker for cancers [46, 47]. *CEP55* expression was positively related to TMB in ACC, PAAD, LGG, SARC, and BRCA. On the contrary, the expression levels of *CEP55* in THYM, KIRP, THCA, and UVM were negatively correlated with TMB (Fig. 5B).

A microsatellite is a short type of tandem repeat DNA train consisting of one to ten nucleotides [48]. *CEP55* expression demonstrated a positive correlation with MSI in six cancers—TGCT, SARC, LUSC, OV, KIRC, and BRCA—and a negative relevance with PRAD, HNSCC, and THCA (Fig. 5C).

A few somatic mutations in tumor DNA can generate immunogenic neoantigens, and these immunogenic peptides can recognize the immune system and target activated T cells [47]. The finding of a correlation of *CEP55* expression with TMB and MSI prompted an investigation of the relationship between *CEP55* expression and neoantigens. The results revealed a weak correlation between *CEP55* expression and neoantigen counts in four tumors (ACC, LUAD, COAD, and PRAD) (Fig. 5D).

**Fig. 6:**
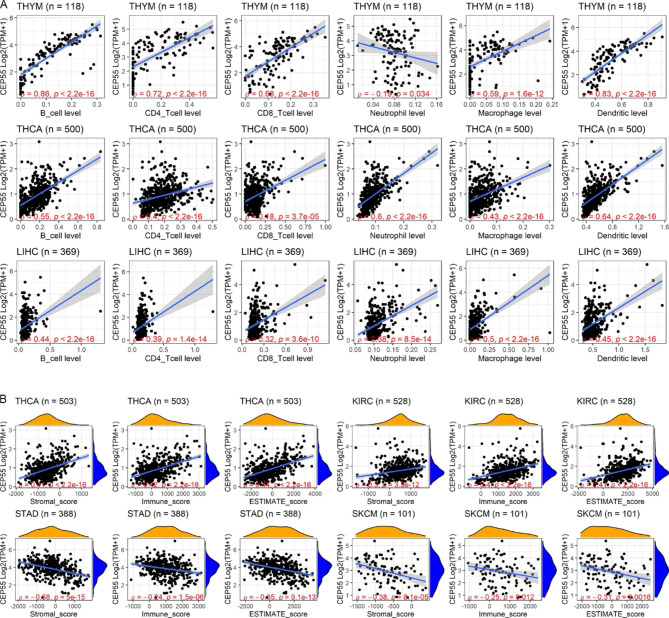
Relation of *CEP55* expression and infiltration levels of immune cells. Panel **A**: TIMER algorithm; **B**: ESTIMATE algorithm

The immune response plays a vital role in antitumor function; therefore, the immune correlation of *CEP55* in pan-cancer was also explored. The TIMER algorithm demonstrated that *CEP55* expression levels tended to positively correlate (except for neutrophils in THYM) with increased infiltration levels of six types of immune cells (especially for B cells, macrophages, and dendritic cells) in THYM, THCA, and LIHC (*ρ* > 0.3, *p* < 0.05; Fig. 6A). Furthermore, based on the ESTIMATE algorithm, a moderate positive relationship between *CEP55* expression and scores of stromal, immune, and ESTIMATE was detected in THCA and KIRC, while a weak negative correlation between *CEP55* expression and the three scores in STAD and SKCM was observed (*p* < 0.05; Fig. 6B).

### Mechanistic prediction of ***CEP55*** in multiple tumors

**Fig. 7:**
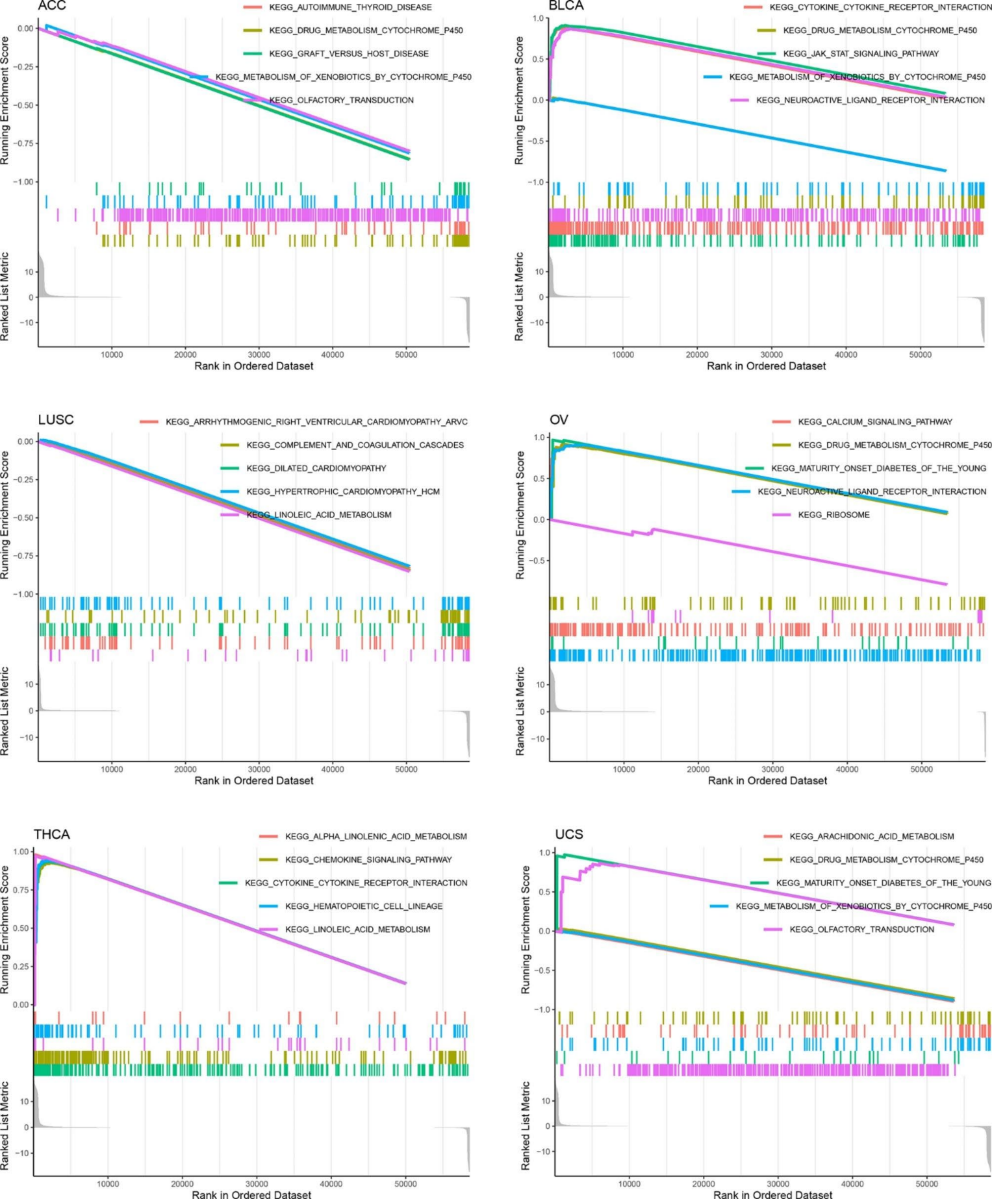
Signaling pathways potentially affected by *CEP55* in multiple cancers

GSEA was performed in this research to explore the underlying mechanisms of *CEP55* in multiple tumors. The peak appeared in the high-expression group, indicating a positive correlation between these pathways and *CEP55* expression, and that these signaling pathways were more active when *CEP55* expression was elevated. *CEP55* expression was correlated with at least five KEGG signaling pathways in six cancers (Fig. 7). Notably, *CEP55* was most likely to affect the “olfactory transduction” pathway in cancers (up to 18 cancer types) and it may could play an important role in “metabolism of xenobiotics by cytochrome P450,” “drug metabolism cytochrome P450,” and “cytokine-cytokine receptor interaction” (Table S3).

### Expression of ***CEP55*** in LUSC

**Fig. 8:**
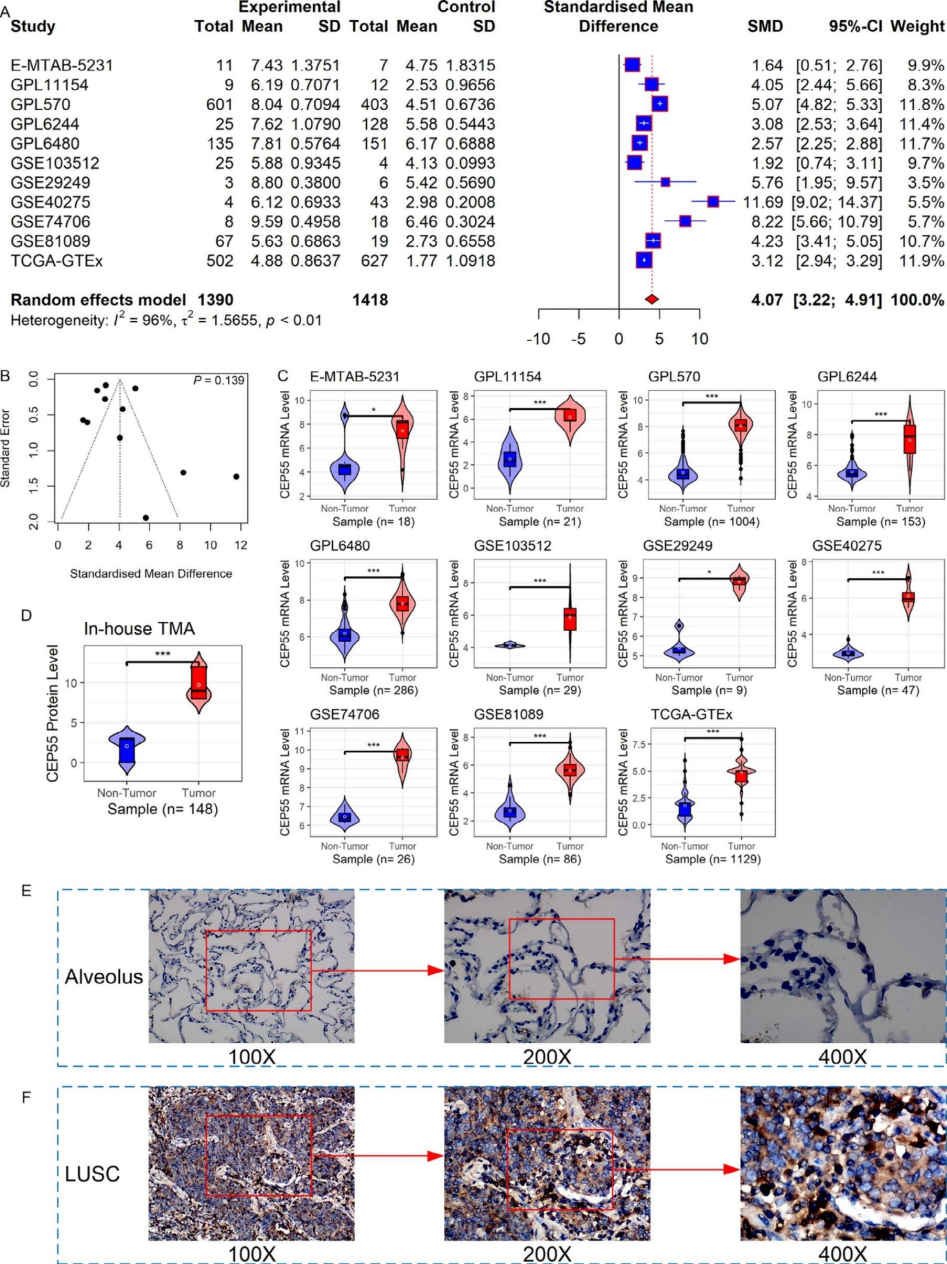
CEP55 mRNA and protein levels in LUSC. Panel **A**: *CEP55* mRNA expression forest plot in LUSC and control tissues. Panel **B**: *Begg*’s test for SMD results. Panel **C**: Violin plots of *CEP55* mRNA expression in each dataset. Panel **D**: The violin plot of CEP55 protein levels. Panels **E**–**F**: Microscopic images of CEP55 protein levels in non-LUSC (**E**) and LUSC (**F**) tissues; the value (e.g., 100X) at the bottom of each microscope image represents the magnification scale of the microscope. The *p*-value was calculated based on the Wilcoxon rank-sum test. ^*^p < 0.05; ^***^p < 0.001

The differential expression and important clinical value of *CEP55* have been observed in several cancers, but little is known about the gene in LUSC. Therefore, using multi-center data, a comprehensive investigation of *CEP55* expression in LUSC was performed in this research. The random effects model showed elevated *CEP55* mRNA expression in the LUSC group but not in the non-LUSC group (SMD = 4.07, 95% CI: 3.22–4.91) (Fig. 8A), and no significant publication bias was detected (*p* > 0.1; Fig. 8B). Regarding each dataset of the 11 merged datasets, the LUSC group showed increased levels of *CEP55* mRNA expression compared with the non-LUSC group (*p* < 0.05; Fig. 8C).

An in-house IHC experiment conducted to verify the CEP55 expression in LUSC at protein levels revealed substantially higher CEP55 protein levels in LUSC tissues than in non-LUSC tissues (*p* < 0.05; Fig. 8D), consistent with the *CEP55* expression at the mRNA levels. Positive CEP55 protein staining was clearly observed by microscopy in LUSC tissues but not in control tissues (Fig. 8E and F).

### Clinical relevance of ***CEP55*** expression in LUSC

**Fig. 9:**
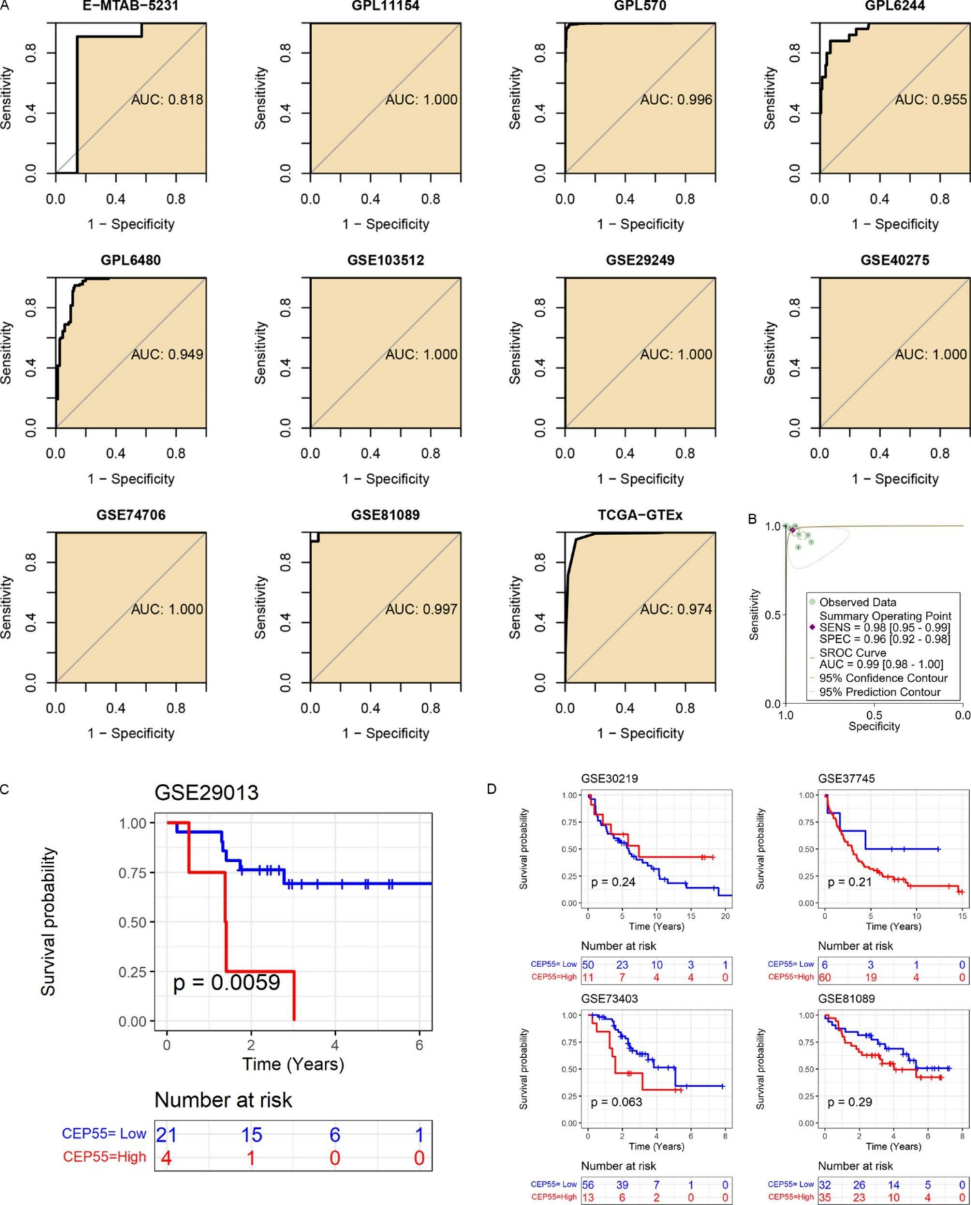
Clinical value of *CEP55* expression in LUSC. Panels **A**–**B**: *CEP55* distinguishes LUSC well from control tissues. Panels **C**–**D**: The correlation of *CEP55* expression with the overall survival of LUSC patients; the red curve represents the high-*CEP55* expression group, while the blue curve represents the low-*CEP55* expression group

As shown in Fig. 9A, ROC curves confirmed the ability of *CEP55* mRNA expression to distinguish LUSC samples from control samples with high accuracy (AUC > 0.8), similar to the findings from the analysis of various cancers. Moreover, the sROC analysis revealed that SCLC samples could be distinguished from non-SCLC samples based on *CEP55* expression (AUC > 0.95, Fig. 9B). These results demonstrate the conspicuous potential for using *CEP55* expression to distinguish LUSC patients from individuals without LUSC.

As shown in Fig. 9C, high *CEP55* mRNA expression was correlated with unfavorable OS. In detail, LUSC patients with overexpression of *CEP55* had more pessimistic OS. The statistically significant result was evident in the GSE29013 (*p* < 0.05; Fig. 9C), while the remaining four cohorts also suggested a trend to an adverse risk associated with *CEP55* expression in the prognosis of LUSC patients (Fig. 9D).

## Discussion

To our knowledge, previous comprehensive research is lacking regarding the expression, clinical value, and potential mechanisms of *CEP55* in pan-cancer, making the present report the first relevant study. The essential roles of *CEP55* in multiple cancers were identified using a CRISPR dataset of 1,068 specimens. Overall, the findings from 11,799 samples of various cancers revealed elevated CEP55 expression levels in 20 cancers. Overexpression of *CEP55* mRNA was a significant predictive and prognostic indicator in various cancers. The expression of *CEP55* was relevant to TMB, MSI, neoantigen counts, and immune cell infiltration in a variety of cancers, indicating that the gene may have an essential role in the immune response. Taking LUSC as an example, the results of this study partly verified the expression level and clinical relevance of CEP55 in cancers based on the analysis of internal tissue microarray and multi-center LUSC data (*n* of samples = 2,956). In conclusion, *CEP55* may be an immune-related predictive and prognostic marker for multiple cancers, including LUSC.

The Chronos scores indicated that, *CEP55* is essential for various tumors originating from some organs, based on the comprehensive pan-cancer analysis of *CEP55* performed in this study. Differential expression of *CEP55* is common in cancer, and its elevated expression has been reported several times. For example, Hauptman et al. [49] demonstrated the upregulation of *CEP55* in colorectal cancer using in-house clinical samples and public data. Yang et al. [9] determined overexpression of *CEP55* in LIHC and attributed it to DNA hypomethylation. Jiang et al. [50] identified elevated *CEP55* expression in non-small-cell lung cancer using 203 specimens. However, no previous investigation has explored *CEP55* expression in pan-cancer. Therefore, our study attempted to fill this void, and its findings revealed upregulated *CEP55* expression in 20 cancers (e.g., BLCA). High *CEP55* expression has also been verified previously in BRAC, CESC, ESAC, and KIRC [51–54]. Our study results also identified overexpression of *CEP55* in CHOL, KICH, KIRP, PRAD, and UCEC. The distinct *CEP55* expression between cancers and controls implies that *CEP55* may have underlying clinical significance in an extensive range of cancers.

Our findings identify *CEP55* expression as a predictor for determining cancer status and indicate a relationship with the prognosis of several cancers. *CEP55* expression provided the ability to distinguish specific cancer tissues (KICH, etc.) from control tissues with high accuracy. To the best of our knowledge, this result has not been previously reported, demonstrating the novelty of our study. To date, previous studies have investigated the prognosis value of *CEP55* expression in some cancers, including ACC, BRCA, GBM, KIRC, LIHC, LUAD, and PAAD [9, 11, 14, 55–58]. The inclusion of more than just these cancers in our study allowed for an analysis of the prognostic value of *CEP55* expression in 33 tumors. Based on our study findings, *CEP55* expression is an OS risk factor for certain cancers (ACC, KICH, KIRC, KIRP, LGG, LIHC, LUAD, MESO, PAAD, PRAD, and UVM) and a protective factor for BRCA and THYM. For DSS, *CEP55* can also be a risk factor for 12 cancers (ACC, etc.). Furthermore, high expression of *CEP55* indicated an unfavorable DFI for patients with KIRP, LIHC, LUAD, PAAD, SARC, and THCA, as well as poor PFI for those diagnosed with one of 14 cancers: ACC, ESCA, KICH, KIRC, KIRP, LGG, LIHC, LUAD, MESO, PAAD, PCPG, PRAD, SARC, and UVM. Overall, *CEP55* may represent a predictive and prognostic marker for multiple cancers.

*CEP55* may have different effects on the immune microenvironment in different cancers. On the one hand, *CEP55* may participate in the negative immune repose in certain cancers (e.g., STAD, and SKCM), since it shows an adverse relationship with the immune microenvironment in these cancers. On the other hand, *CEP55* may present an immune response activation factor for the following reasons. First, high levels of TMB and MSI frequently promote the occurrence of neoantigens, and neoantigens tend to stimulate an immune response [46]. Notably, based on our study results, *CEP55* was not only positively related to TMB and MSI in several cancers (SARC, etc.) but it was also positively related to neoantigens in certain cancers (ACC, etc.). Second, dendritic cells are often considered the most functional professional antigen-presenting cells in the adaptive immune response of humans, responsible for transporting tumor antigens and activating antitumor T cells [59]. Interestingly, *CEP55* expression was positively correlated with six immune cell types (not only dendritic cells, but also B cells, CD4 + T cells, CD8 + T cells, neutrophils, and macrophages) in specific cancers, namely — THYM, THCA, and LIHC (except for neutrophils in THYM). Third, a weak positive correlation was detected between *CEP55* expression and three immune scores in certain cancers (e.g., THCA and KIRC). Thus, our study results indicated a close relationship between *CEP55* expression and the immune environment. However, the current findings require further experimental validation.

The underlying mechanisms of *CEP55* in various cancers remain complex; for example, *CEP55* expression was correlated with at least five KEGG signaling pathways in six cancers (ACC, etc.). Some signaling pathways may be critical for *CEP55* involvement in multiple cancers, including “olfactory transduction,” “metabolism of xenobiotics by cytochrome P450,” “drug metabolism cytochrome P450,” and “cytokine-cytokine receptor interaction” signaling pathways. Among these signaling pathways, the *CEP55*-affected activation of “cytokine-cytokine receptor interaction” signaling pathway directly highlights the association between *CEP55* expression and immune cell infiltration levels to some extent. Cytokines are secreted proteins primarily produced by various cell types, particularly immune cells, and some of them play a vital role in the antitumor process [60, 61]. During the antitumor process, immune cells are activated and produce cytokines. When these cytokines bind to their respective receptors, they transmit signals between cells and regulate various biological processes, including cell growth, differentiation, proliferation, and cell death. Notably, certain cytokines such as IL-2 and IL-15 can influence the proliferation of immune cells, thereby contributing to the regulation of immune cell infiltration levels [60, 62]. Thus, a correlation exists between the level of immune cell infiltration and some signaling pathways (at least the “cytokine-cytokine receptor interaction” signaling pathway). Considering the association of *CEP55* expression with immune cell infiltration and the potential activation status of the “cytokine-cytokine receptor interaction” signaling pathway, *CEP55* may play an important role in tumors through its effects on these two factors. Taken together, our study results provide a clue for further experimental explorations into the possible molecular mechanisms of *CEP55* in pan-cancer.

With the view that *CEP55* is likely to play a vital role in various cancers, an attempt was made to validate the expression and clinical significance of *CEP55* in one specific tumor type (i.e., LUSC). Previously, Fu et al. [63] and Shi et al. [64] investigated *CEP55* in LUSC; however, they included small-sized samples (*n* < 200), and they did not verify their results with multi-center and internal samples, raising the possibility of some limitations. For instance, Fu et al. [63] did not find an association of *CEP55* with the prognosis of patients with LUSC, which was identified in our study in one of the five cohorts; this also indicates an advantage of studies based on multiple cohorts. Notably, the use of internal tissue microarrays and multi-center LUSC data in our study revealed elevated CEP55 mRNA and protein levels in LUSC. Furthermore, *CEP55* could identify LUSC, and high *CEP55* expression was associated with poor prognosis in LUSC patients, which partly supports the results in the pan-cancer analysis.

Our study had some limitations. The number of body-fluid samples used in this study could be expanded, as this would facilitate the validation of the mRNA and protein levels of CEP55 in pan-cancer analysis and the correlation between *CEP55* and prognosis. A need remains to include in vivo and in vitro experiments to support the study of the molecular mechanisms of *CEP55* in multiple cancer cells. Future studies should include internal samples from multiple cancers, not just from LUSC.

In conclusion, our study provides findings that offer a comprehensive assessment of *CEP55* and identify the significant clinical value of *CEP55* in pan-cancer. *CEP55* may be an immune-related predictive and prognostic marker for certain cancers, including LUSC.

## Electronic supplementary material

Below is the link to the electronic supplementary material.

Supplementary Material 1

Supplementary Material 2

Supplementary Material 3

Supplementary Material 4

Supplementary Material 5

Supplementary Material 6

## Data Availability

Public data supporting the findings of this study are openly available from the Depmap Portal (https://depmap.org/portal/), TCGA (https://tcga-data.nci.nih.gov/), ArrayExpress, GEO (http://www.ncbi.nlm.nih.gov/geo/), GTEx (https://gtexportal.org/), CPTAC (https://pdc.cancer.gov/pdc/), TIMER (https://cistrome.shinyapps.io/timer/), and SangerBox (v3.0) databases. Data collected from the SangerBox (v3.0) are stored in GitHub (https://github.com/Guosheng-Li/CEP55-LUSC-2023). The datasets used in this study include E-MTAB-5231, GSE29249, GSE103512, GSE74706, GSE40275, GSE81089, GSE84776, GSE70089, GSE19188, GSE19804, GSE30219, GSE18842, GSE101929, GSE157010, GSE106937, GSE18385, GSE33532, GSE50081, GSE43580, GSE37745, GSE28571, GSE29013, GSE10245, GSE2109, GSE27556, GSE31552, GSE44077, GSE51852, GSE33479, GSE101420, GSE73403, GSE40074, GSE40588, PDC000121, PDC000204, PDC000221, PDC000127, PDC000219, PDC000270, PDC000125, TCGA-LUSC, and GTEx. In-house data are available from the correspondent for reasonable applications.

## List of abbreviations

AJCC: American Joint Committee on Cancer
AUC: area under the curve
BLCA: bladder urothelial carcinoma
BRCA: breast invasive carcinoma
CEP55: centrosomal protein 55
CHOL: cholangiocarcinoma
COAD: colon adenocarcinoma
CESC: cervical squamous cell carcinoma and endocervical adenocarcinoma
CI: confidence interval
DFI: disease-free interval
DSS: disease-specific survival
ESCA: esophageal carcinoma
GBM: glioblastoma multiforme
GSEA: gene set enrichment analysis
HNSCC: head and neck squamous cell carcinoma
HR: hazard ratio
IHC: immunohistochemistry
KEGG: Kyoto Encyclopedia of Genes and Genomes
KICH: kidney chromophobe
KIRC: kidney renal clear cell carcinoma
KIRP: kidney renal papillary cell carcinoma
LIHC: liver hepatocellular carcinoma
LUAD: lung adenocarcinoma
LUSC: lung squamous cell carcinoma
mRNA: messenger RNA
PAAD: pancreatic adenocarcinoma
PCPG: pheochromocytoma and paraganglioma
PFI: progression-free interval
PRAD: prostate adenocarcinoma
READ: rectum adenocarcinoma
ROC: receiver operating characteristic
sROC: summary receiver operating characteristic
SMD: standardized mean difference
STAD: stomach adenocarcinoma
TCGA: The Cancer Genome Atlas
TMB: tumor mutation burden
MSI: microsatellite instability
THCA: thyroid carcinoma
UCEC: uterine corpus endometrial carcinoma
ACC: adrenocortical carcinoma
DLBC: lymphoid neoplasm diffuse large b-cell lymphoma
LAML: acute myeloid leukemia
LGG: brain lower grade glioma
MESO: mesothelioma
OS: overall survival
OV: ovarian serous cystadenocarcinoma
TGCT: testicular germ cell tumors
UCS: uterine carcinosarcoma
UVM: uveal melanoma
SKCM: skin cutaneous melanoma
SARC: sarcoma
THYM: thymoma
