# Supplementary material for: CEP55: an immune-related predictive and prognostic molecular biomarker for multiple cancers
Source: BMC Pulm Med. 2023 May 12;23:166. doi: 10.1186/s12890-023-02452-1 (PMC10182662; doi:10.1186/s12890-023-02452-1)
Supplement: Supplementary file 1 — Supplementary Material 1 [file 12890_2023_2452_MOESM1_ESM.docx]

**Table S1**. Cancers and their samples included in this study.

| **Cancer** | **Tumor number** | **Control number** |
| --- | --- | --- |
| Bladder urothelial carcinoma (BLCA) | 407 | 19 |
| Breast invasive carcinoma (BRCA) | 1092 | 113 |
| Cholangiocarcinoma (CHOL) | 36 | 9 |
| Colon adenocarcinoma (COAD) | 288 | 41 |
| Cervical squamous cell carcinoma and endocervical adenocarcinoma (CESC) | 304 | 3 |
| Esophageal carcinoma (ESCA) | 181 | 13 |
| Glioblastoma multiforme (GBM) | 153 | 5 |
| Head and neck squamous cell carcinoma (HNSCC) | 518 | 44 |
| Kidney chromophobe (KICH) | 66 | 25 |
| Kidney renal clear cell carcinoma (KIRC) | 530 | 72 |
| Kidney renal papillary cell carcinoma (KICP) | 288 | 32 |
| Liver hepatocellular carcinoma (LIHC) | 369 | 50 |
| Lung adenocarcinoma (LUAD) | 513 | 59 |
| Lung squamous cell carcinoma (LUSC) | 498 | 50 |
| Pancreatic adenocarcinoma (PAAD) | 178 | 4 |
| Pheochromocytoma and paraganglioma (PCPG) | 177 | 3 |
| Prostate adenocarcinoma (PRAD) | 495 | 52 |
| Rectum adenocarcinoma (READ) | 92 | 10 |
| Stomach adenocarcinoma (STAD) | 414 | 36 |
| Thyroid carcinoma (THCA) | 504 | 59 |
| Uterine corpus endometrial carcinoma (UCEC) | 180 | 23 |
| Adrenocortical carcinoma (ACC) | 77 | 0 |
| Lymphoid neoplasm diffuse large B-cell lymphoma (DLBC) | 47 | 0 |
| Acute myeloid leukemia (LAML) | 173 | 0 |
| Brain lower grade glioma (LGG) | 509 | 0 |
| Mesothelioma (MESO) | 87 | 0 |
| Ovarian serous cystadenocarcinoma (OV) | 419 | 0 |
| Testicular germ cell tumors (TGCT) | 148 | 0 |
| Uterine carcinosarcoma (UCS) | 57 | 0 |
| Uveal melanoma (UVM) | 79 | 0 |
| Skin cutaneous melanoma (SKCM) | 102 | 0 |
| Sarcoma (SARC) | 258 | 0 |
| Thymoma (THYM) | 119 | 0 |
| Total | 9358 | 722 |
