# Supplementary material for: CEP55: an immune-related predictive and prognostic molecular biomarker for multiple cancers
Source: BMC Pulm Med. 2023 May 12;23:166. doi: 10.1186/s12890-023-02452-1 (PMC10182662; doi:10.1186/s12890-023-02452-1)
Supplement: Supplementary file 2 — Supplementary Material 2 [file 12890_2023_2452_MOESM2_ESM.docx]

**Table S2.** LUSC-related datasets and their samples included in this study.

| Datasets | Platform | Sample_number (LUSC vs. control) |
| --- | --- | --- |
| E-MTAB-5231 | HG-U133_Plus_2 | 11 vs. 7 |
| GSE29249 | GPL10558 | 3 vs. 6 |
| GSE103512 | GPL13158 | 25 vs. 4 |
| GSE74706 | GPL13497 | 8 vs. 18 |
| GSE40275 | GPL15974 | 4 vs. 43 |
| GSE81089 | GPL16791 | 67 vs. 19 |
| GSE84776 | GPL11154 | 9 vs. 9 |
| GSE70089 | GPL11154 | 0 vs. 3 |
| GSE19188 | GPL570 | 27 vs. 65 |
| GSE19804 | GPL570 | 0 vs. 60 |
| GSE30219 | GPL570 | 61 vs. 14 |
| GSE18842 | GPL570 | 0 vs. 45 |
| GSE101929 | GPL570 | 0 vs. 34 |
| GSE157010 | GPL570 | 235 vs. 0 |
| GSE106937 | GPL570 | 1 vs. 4 |
| GSE18385 | GPL570 | 0 vs. 161 |
| GSE33532 | GPL570 | 16 vs. 20 |
| GSE50081 | GPL570 | 43 vs. 0 |
| GSE43580 | GPL570 | 73 vs. 0 |
| GSE37745 | GPL570 | 66 vs. 0 |
| GSE28571 | GPL570 | 28 vs. 0 |
| GSE29013 | GPL570 | 25 vs. 0 |
| GSE10245 | GPL570 | 18 vs. 0 |
| GSE2109 | GPL570 | 6 vs. 0 |
| GSE27556 | GPL570 | 2 vs. 0 |
| GSE31552 | GPL6244 | 25 vs. 62 |
| GSE44077 | GPL6244 | 0 vs. 66 |
| GSE51852 | GPL6480 | 28 vs. 4 |
| GSE33479 | GPL6480 | 14 vs. 27 |
| GSE101420 | GPL6480 | 0 vs. 60 |
| GSE73403 | GPL6480 | 69 vs. 0 |
| GSE40074 | GPL6480 | 24 vs. 0 |
| GSE40588 | GPL6480 | 0 vs. 60 |
| TCGA | TCGA-GTEx | 502 vs. 49 |
| GTEx | TCGA-GTEx | 0 vs. 578 |
| Total | / | 1390 vs. 1418 |
