# Supplementary material for: CEP55: an immune-related predictive and prognostic molecular biomarker for multiple cancers
Source: BMC Pulm Med. 2023 May 12;23:166. doi: 10.1186/s12890-023-02452-1 (PMC10182662; doi:10.1186/s12890-023-02452-1)
Supplement: Supplementary file 3 — Supplementary Material 3 [file 12890_2023_2452_MOESM3_ESM.docx]

**Table S3.** The KEGG signaling pathways that CEP55 may participate in cancers.

| KEGG signaling pathway | Number of pathways-related cancers |
| --- | --- |
| Olfactory transduction | 18 |
| Metabolism of xenobiotics by cytochrome P450 | 7 |
| Drug metabolism cytochrome P450 | 5 |
| Cytokine cytokine receptor interaction | 4 |
| Maturity onset diabetes of the young | 4 |
| Retinol metabolism | 4 |
| Ribosome | 4 |
| Autoimmune thyroid disease | 3 |
| Calcium signaling pathway | 3 |
| Cell cycle | 3 |
| Jak stat signaling pathway | 3 |
| Linoleic acid metabolism | 3 |
| Allograft rejection | 2 |
| Arachidonic acid metabolism | 2 |
| Asthma | 2 |
| Dilated cardiomyopathy | 2 |
| Graft versus host disease | 2 |
| Neuroactive ligand receptor interaction | 2 |
| Pentose and glucuronate interconversions | 2 |
| Aldosterone regulated sodium reabsorption | 1 |
| Alpha linolenic acid metabolism | 1 |
| Arrhythmogenic right ventricular cardiomyopathy arvc | 1 |
| Ascorbate and aldarate metabolism | 1 |
| Chemokine signaling pathway | 1 |
| Complement and coagulation cascades | 1 |
| Hedgehog signaling pathway | 1 |
| Hematopoietic cell lineage | 1 |
| Hypertrophic cardiomyopathy hcm | 1 |
| Natural killer cell mediated cytotoxicity | 1 |
| Phenylalanine metabolism | 1 |
| Primary immunodeficiency | 1 |
| Taste transduction | 1 |
