# Supplementary figures and images for: CEP55: an immune-related predictive and prognostic molecular biomarker for multiple cancers
Source: BMC Pulm Med. 2023 May 12;23:166. doi: 10.1186/s12890-023-02452-1 (PMC10182662; doi:10.1186/s12890-023-02452-1)

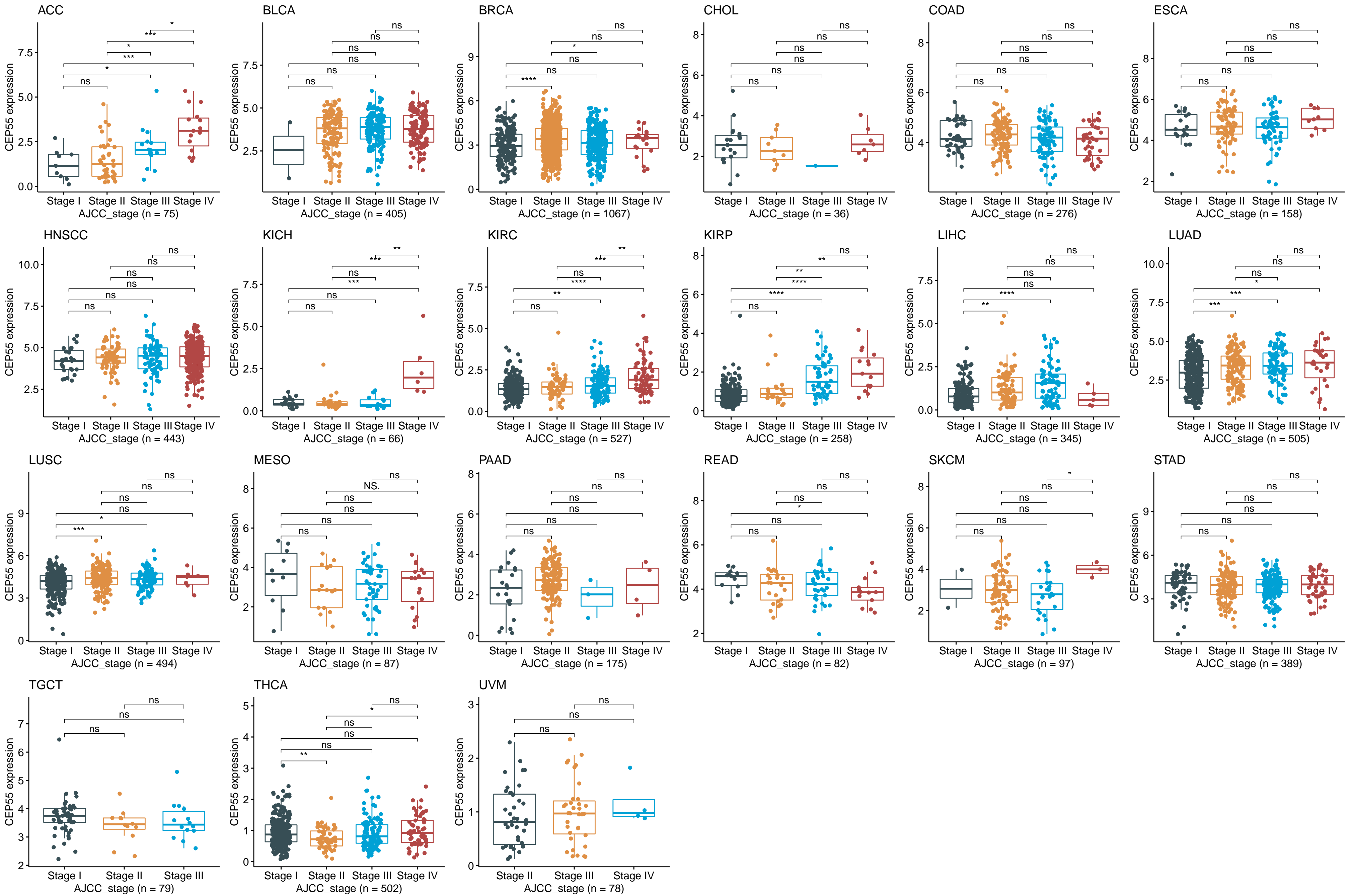

Figure S1. The difference in CEP55 expression between cancer patients of tumor stages.

Supplement: Supplementary file 4 — Supplementary Material 4 [file 12890_2023_2452_MOESM4_ESM.pdf]

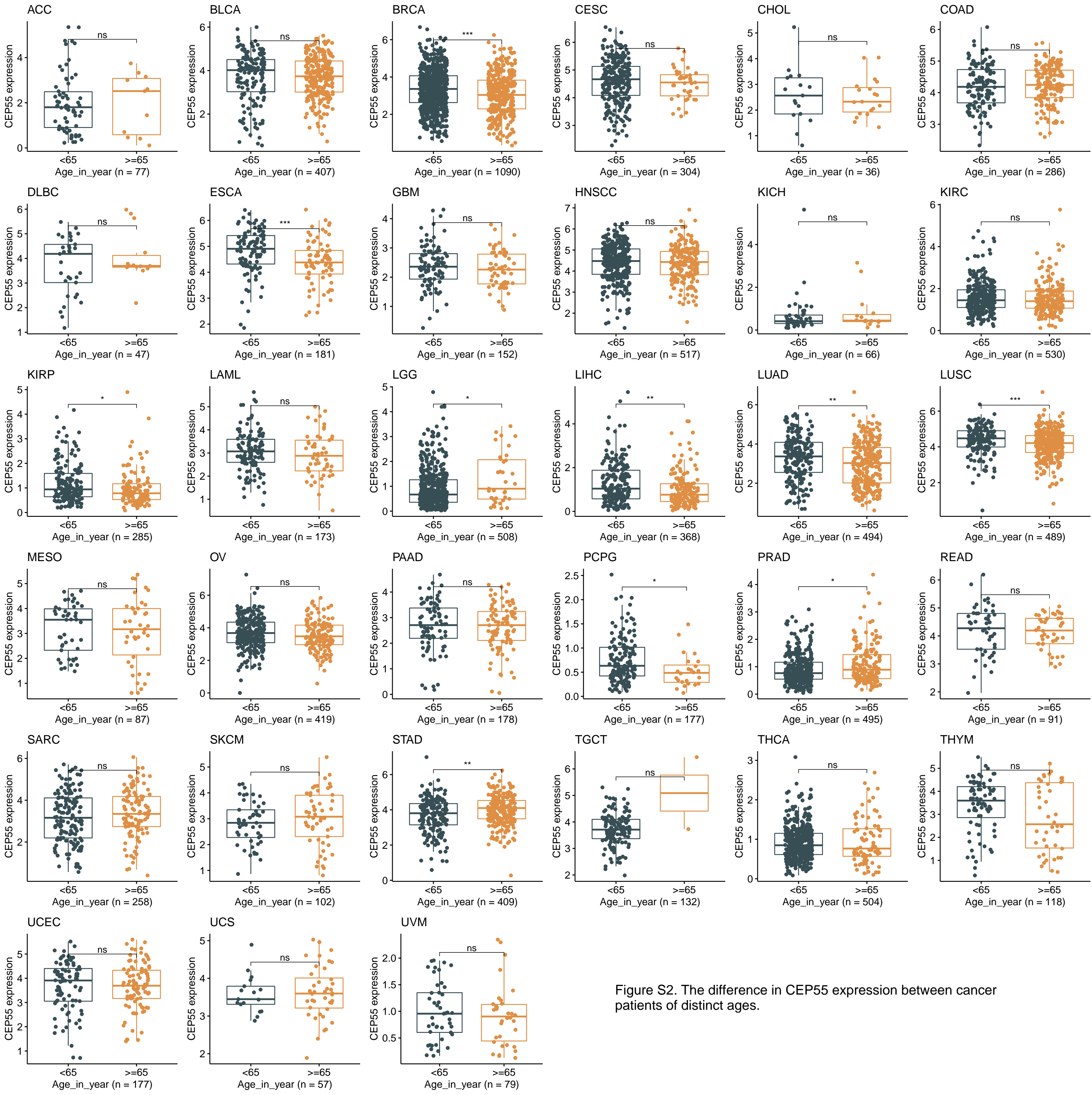

Figure S2. The difference in CEP55 expression between cancer patients of distinct ages.

Supplement: Supplementary file 5 — Supplementary Material 5 [file 12890_2023_2452_MOESM5_ESM.pdf]

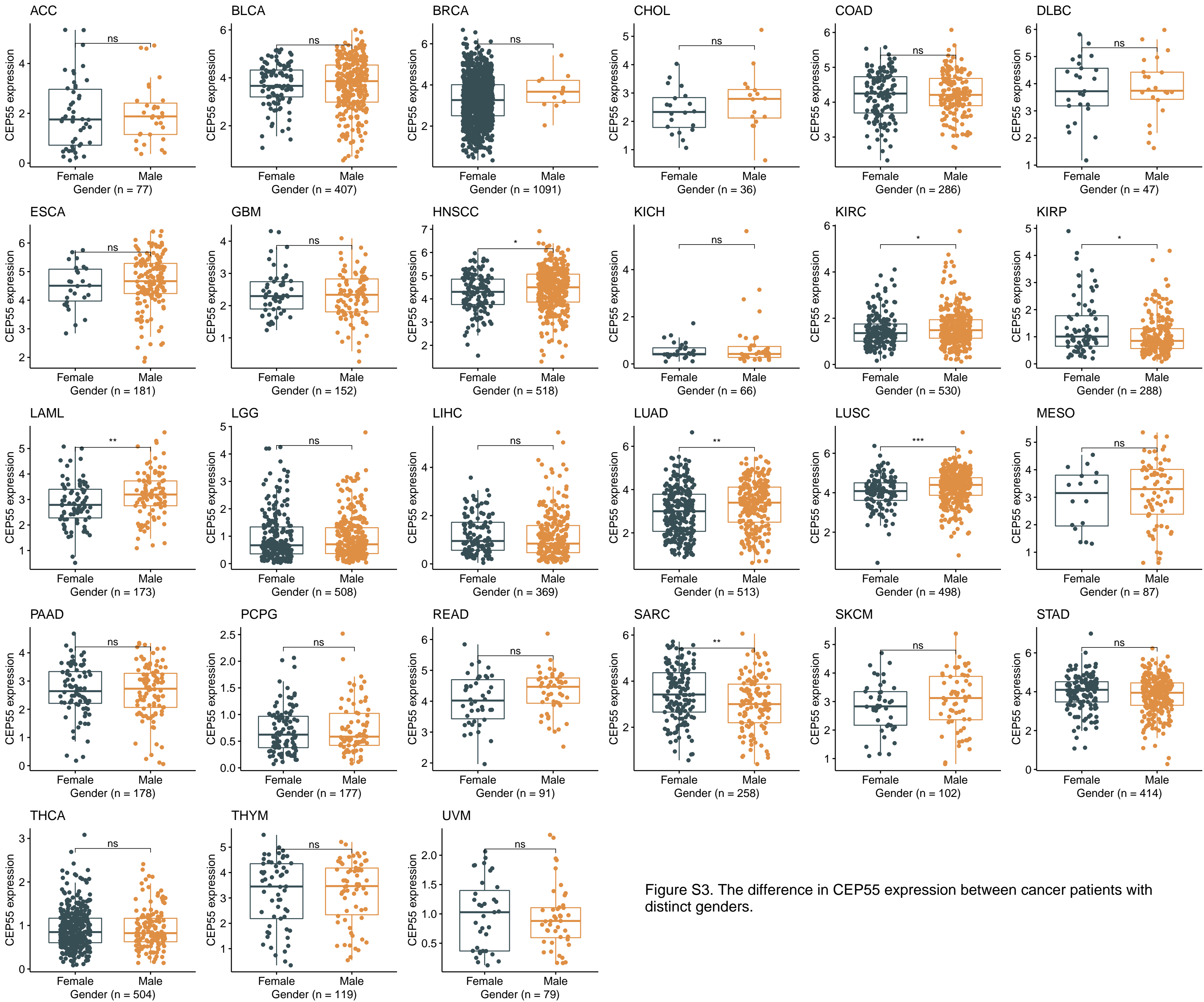

Figure S3. The difference in CEP55 expression between cancer patients with distinct genders.

Supplement: Supplementary file 6 — Supplementary Material 6 [file 12890_2023_2452_MOESM6_ESM.pdf]
